# The impact of dietary preference on household food waste: evidence from China

**DOI:** 10.3389/fnut.2024.1415734

**Published:** 2024-07-09

**Authors:** Li Zhang, Linxiang Ye, Long Qian, Xiuping Zuo

**Affiliations:** ^1^Institute of Food and Strategic Reserves, Nanjing University of Finance and Economics, Nanjing, China; ^2^School of Economics, Nanjing University of Finance and Economics, Nanjing, China; ^3^School of Business, Jiangsu Open University, Nanjing, China

**Keywords:** food waste, dietary preference, refrigerator use, China, carbon footprint

## Abstract

Food waste jeopardizes food security and causes economic and resource losses. Household food waste is the most significant source of global food waste and urgently needs to be reduced. Based on the China Health and Nutrition Survey (CHNS), our study estimates the daily food waste data of 6,418 sample observations across China and the dietary preference scores of their household heads. Using a count regression model, our study explores the relationship between dietary preference and household food waste in Chinese households, and further explores the moderating function of household refrigerator use. The study has found that: (1) improving dietary preference score can significantly reduce household food waste ratio, and robustness tests support this finding. (2) There is a positive moderating effect of refrigerator use in the process of how dietary preference influence food waste. (3) Heterogeneity analysis shows that the impact of dietary preference on household food waste varies by gender and age of the household head, household size, economic level, urban–rural type, and north–south region. Our study provides evidence that improving dietary preference can reduce household food waste in China, which has certain implications for waste reduction in other developing countries.

## 1 Introduction

Food waste not only affects global food security, leading to the wastage of production resources, but also imposes a huge burden on the environment (1–3). According to the United Nations Environment Programme Report, the total global food waste in 2022 will amount to 1,052 million tonnes, with 132 kilograms of food wasted *per capita*, which is one-fifth of the total amount of food available to consumers (4). Meanwhile, one out of every nine people in the global population is suffering from hunger (5). If this food is effectively utilized, it will contribute to the eradication of global hunger and malnutrition (6). Furthermore, food waste implies the ineffective depletion of factor resources such as water, soil, and fertilizers invested in the production stage (7, 8). It is estimated that food losses and wastage take up about 24% of the total global utilization of land for food production, freshwater resources and fertilizers (9). Moreover, food waste globally generates 3.3 gigatons of carbon dioxide, an equivalent of 7% of total greenhouse gas (GHG) emissions causing climate change (10–12). Therefore, how to effectively reduce food waste has become a crucial theoretical and practical issue.

As an international issue, food waste has attracted the attention of many scholars and the related results are abundant (13). According to the United Nations Environment Programme (UNEP), Canada wastes 189 kg of food *per capita* per year, and the United States wastes 159 kg of food *per capita* per year (UNEP, 2024). Gilbert and Ricci found that 94 kg of food is wasted per year in a study of Rio de Janeiro, Brazil (4). Xue et al. found that 29 kg of food is wasted per year in China (4). The research on the structure of food waste indicates that with much of the waste concentrates on foods such as grains, fruits, and vegetables. Conrad (14) finds in a study on the United States that fruits and vegetables are the main categories of food waste in households. Caldeira et al. (15) finds that grains, fruits, and vegetables are the most wasted foods in households. Ammann et al. (16) and Ananda et al. (17) also indicate that fruits and vegetables account for the highest proportion of household food waste. Li et al. (18) uses Logit and Tobit models to estimate the relationship between five food categories and consumer characteristics in China, and concludes that the incidence and proportion of fruit and vegetable waste are the highest. In terms of economic loss, Australia loses $20 billion annually due to food waste (19). 58% of all food produced is lost or wasted in Canada, at a cost of $49 billion (20). In addition, the economic loss from food waste in the household sector alone is 0.82% of South Africa’s GDP (21, 22).

It can be seen that developed countries are the most significant contributors to global food waste (23). The existing researches are mostly from developed countries such as the United Kingdom, the Netherlands, the United States, Portugal, Germany, and Canada, with relatively few studies on emerging developing countries such as China (11). However, as a developing country with a population of over 1.4 billion, China is also experiencing an increasing trend of food waste due to the improvement of living standards. The total amount of food waste in China is about 120 million tons per year (24). The economic loss caused by food loss and waste is up to $263.55 billion in China (25, 26). The study of food waste and its influencing factors in China is of great significance for reducing global food waste and ensuring food security.

This paper endeavors to study the problem of food waste in Chinese households. The China Health and Nutrition Survey (CHNS) database uses questionnaires to collect data. The questionnaires design includes questions on individual, household, community and dietary surveys to obtain detailed and specific information. In addition, a count regression model is used for statistical analyses to understand the magnitude of the effect of differences in household members’ dietary preferences on household food waste and the moderating effect of refrigerator use.

From the unique perspective of dietary preference, this study analyses the effect of differences in dietary preferences on household food waste in China and suggests countermeasures. This study not only enriches the research on the influencing factors of household food waste in China, but also provides a reference for emerging developing countries in this field to reduce food waste and ensure food security.

The subsequent organization of our study is as follows: the second part is literature review. The third part is the materials and methods, including data sources, variable settings and model settings. The fourth part is the results. The fifth part is the discussion. The last part is the conclusions.

## 2 Literature review

Household food waste is generated in a variety of ways during the process of consuming food in households (27). There are a wealth of researches on this area, with existing studies focusing on both consumer behaviors and social practices. The theory of consumer behavior assumes that individual behavior is determined by the intention to execute the behavior. On the one hand, the influence of intention is manifested in the different demographic, psychological, and habitual factors that affect household food waste differently (28). Specifically, the factors are the age of the head of the household (29), literacy (30), religious beliefs (31), the demographic structure of the household (2), the level of household income (32), social capital (33), famine experience (34), motivation for veganism (18), body image management needs (11), food purchasing and preparation (35), processing and cooking techniques (36), food storage habits (37), wasteful habits (38) and more. On the other hand, the effect of intention is reflected in the correlation between individual frugality and environmental awareness (39) and food waste behavior.

The theory of social practice suggests that macro-environments such as cultural, economic, and social contexts, as well as the micro-environments surrounding individuals, influence individual food waste behavior. Tang et al. (40), from the perspective of dietary anthropology, argues that the causes of food waste in the population are the shared meal system, the preference for lavish banquets, and the food culture practice of ‘saving face’. Kansal et al. (23) mentions that the developed countries are the main contributors to food waste, with a higher rate of food waste compared to the developing countries. It can be seen that the level of economic development significantly affects the level of food waste. From the social level, Cheng (28) believes that information intervention positively affects food waste. Porpino et al. (41), Wharton et al. (42), and Yildirim et al. (43) discover from the micro-environment in which an individual lives that compared to small vegetable stores and self-cultivation, choosing to buy food in bulk from large supermarkets leads to more food waste. Secondi et al. (29) argues that the size of utensils also affects food waste generation, with larger plates inducing people to prepare more meals, which in turn increases the amount of food waste.

As the *per capita* income level of Chinese residents increases, the lifestyle and dietary structure have changed considerably. Dietary preference may be an important factor influencing food waste.

With a given amount of household food preparation, on the one hand, consumers with the preference for fast food choose to order food or eat out, leaving home-cooked food wasted due to non-consumption. On the other hand, consumers’ preferences for household-specific foods, such as salty snacks and soft drinks, reduce the consumption of other foods, leaving a large amount of healthful, edible food usually unused or discarded from the household kitchen (27). In particular, children’s selective eating behavior and preference can lead to overpreparation and provision of food by parents, resulting in waste (23). Therefore, it is reasonable to hypothesize that dietary preference is related to what and how much food is wasted. However, there are few studies on the relationship between dietary preference and food waste in Chinese households. For this reason, our study focuses on how dietary preference affect food waste in Chinese households, providing Chinese evidence for understanding and improving dietary preference and then reducing food waste from a global perspective.

## 3 Materials and methods

The research framework is as follows: First, based on the existing CHNS data, we select and clean the samples; Second, we set the variables according to the existing studies; Third, we select the count regression model according to the research needs; Finally, we conduct the statistical analyses.

### 3.1 Data sources

**Figure 1:**
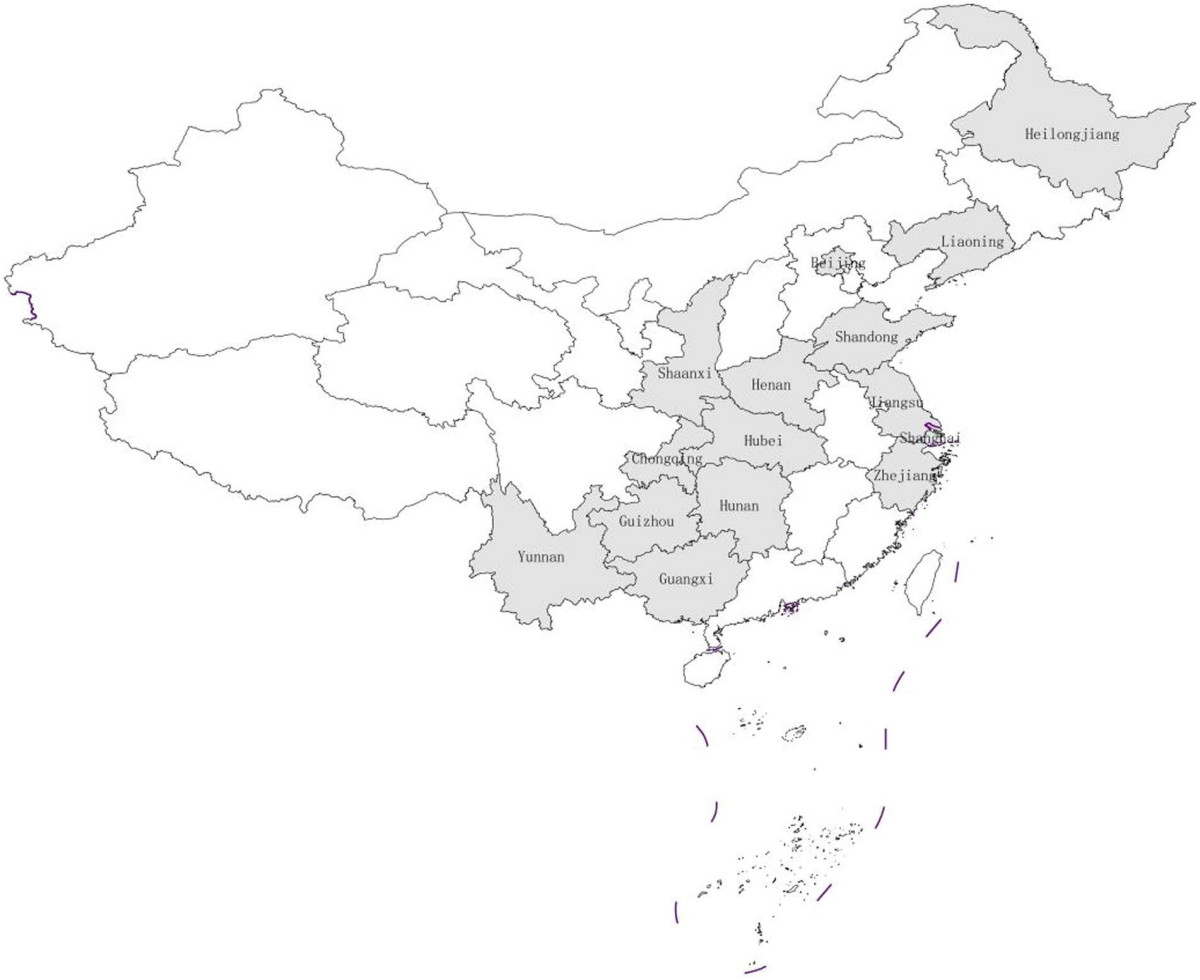
Distribution of CHNS samples.

The data used in our study is from the CHNS, a collaboration between the University of North Carolina and the Chinese Center for Disease Control and Prevention (CDC), which is the only larger-scale micro-database of household food waste in China. The CHNS survey includes data from 10 periods: 1989, 1991, 1993, 1997, 2000, 2004, 2006, 2009, 2011, and 2015, covering the provinces of Beijing, Liaoning, Heilongjiang, Shanghai, Yunnan, Jiangsu, Shandong, Henan, Hubei, Zhejiang, Hunan, Guangxi, Guizhou, Chongqing, and Shaanxi (Figure 1). The CHNS database has been conducting research on respondents’ knowledge of dietary preference since 2004. The latest survey data released is for 2015, but the 2015 data does not include dietary information. Furthermore, the 2011 dietary data only includes consumption in terms of edible oils and condiments. Therefore, our study uses data from 2004, 2006, and 2009, which is an unbalanced panel data study because some survey data is missing.

The data is cleaned as follows: firstly, samples with serious missing information are excluded based on the completeness of information on key variables such as the amount of food waste and the rate of food waste. Secondly, to minimize the interference of anomalous data with the results, samples with a total amount of food preparation for 3 days less than 0 are excluded, and the total amount of food waste and the rate of food waste for 3 days in the household are reduced to the tail at 1 and 99%. Our study ends up with 6,418 observations: 1168 samples in 2004, 1710 samples in 2006, and 3,540 samples in 2009.

### 3.2 Variable settings

#### 3.2.1 Food waste

Any phenomenon that alters the availability, palatability, healthful properties or quality of food, thereby reducing its own value, is collectively referred to as food loss; food loss at the consumption stage is food waste. It can be seen that food waste refers to the food loss that can be avoided in the consumption process under the existing conditions, but the parts that are not suitable for consumption (such as vegetable peels, bean dregs and bones, etc.) are not included in the statistics (44, 45). In the measurement of food waste in residential households, based on the availability of data, scholars mostly regard the amount of food discarded on three consecutive days as the amount of food waste directly (27). Our study also adopts such methods to obtain food waste data. Starting from the correlation between the amount of food waste and the amount of food preparation, our study characterizes the level of food waste by calculating the food waste ratio [food waste ratio = food waste*100/(total food purchase or self-production + stock – total surplus in 3 days)]. It is worth noting that the food items on which household wastage is measured basically cover the food groups consumed by households on a daily basis. There are three categories: cereals, vegetables and meat, eggs and milk.

#### 3.2.2 Dietary preference

Drawing on the research of Min et al. (27), a composite score is calculated using the entropy method based on the respondents’ scores from the food preference questionnaire. The food preference of the household members is measured by five questions on consumption of fast food, savory snacks, fruits, vegetables, and soft drinks as shown in Appendix Table A1. Responses for each food item consist of five options: very dislike, dislike, neutral, like, and like very much, and are assigned a score of 1, 2, 3, 4, and 5 in that order.

The more one likes fast food, snacks and sugary drinks, the higher the score for such foods, which are not conducive to health according to the healthy eating concept. Due to the premise that the higher the food preference score, the healthier the diet of the family members. Therefore, the direction of these three questions is adjusted to be positive. The score of 5 is adjusted to 1. The score of 4 is adjusted to 2. The score of 3 remains unchanged according to the World Health Organisation 1988 criteria. The composite food preference score is calculated based on the adjusted respondents’ scores for the five food preferences according to the entropy weighting method.

#### 3.2.3 Control variables

Referring to the existing literature (27, 34) and based on data availability, multiple variables in the dimensions of household head characteristics, household size and economic characteristics are introduced to mitigate the omitted variable problem as much as possible. Specifically, because the head of household has a decision-making role in household purchases and consumption, the gender, age, education level, ethnicity, employment and labor intensity status of the head of household are controlled (27, 46). Household-level variables include the proportion of meals eaten away from home, household income *per capita*, household size, proportion of household members under 14 years old, proportion over 60 years old, and average BMI of household members (11, 34). In addition, the level of community development, urban–rural type (27) are also introduced to control for the possible effects of community-level factors. Considering the endogeneity issue of omitted variables, our study also controls for temporal and regional characteristics. The definitions and descriptive statistics of the variables are presented in Table 1.

**Table 1:** The definition and statistical analysis of variables.

Variable	Definition	Mean	St. dev.	Min	Max
Food waste ratio	Food waste for three consecutive days * 100/(total food purchase or self-production + stock – total surplus in three days) (%)	3.21	4.47	0.00	22.06
Weight of food waste	Food waste *per capita* in households for three consecutive days (g)	92.54	139.23	0.00	712.50
DP	Mean dietary preference score of household members	3.39	0.53	0.42	4.86
RU	Whether having a refrigerator (1 = yes, 0 = no)	0.63	0.48	0.00	1.00
Gender	1 = male,0 = female	0.81	0.39	0.00	1.00
Age	Age recorded on the day of the survey (years)	53.65	13.06	16.00	94.00
Education	1 = Elementary school and below, 2 = Middle school, 3 = High school or Technical secondary school, 4 = Junior college and above	1.99	0.94	1.00	4.00
Ethnicity	1 = Han, 0 = minority	0.90	0.30	0.00	1.00
Work status	Whether working (1 = yes; 0 = no)	0.60	0.49	0.00	1.00
Working intensity	Distributed from 1 to 5, with intensity increasing in steps	2.45	1.26	1.00	5.00
Proportion of families eating out	Number of meals eaten away from home/total number of meals eaten over 3 days (%)	8.78	18.22	0.00	100.00
Family economic condition	Household income *per capita* (yuan, log)	8.62	1.45	0.00	12.61
Household size	Total number of family members (persons)	3.24	1.43	1.00	13.00
Age 14	Proportion of family members aged 14 years and less (%)	9.48	15.80	0.00	75.00
Age 60	Proportion of family members aged 60 years and older (%)	24.84	37.84	0.00	100.00
Family BIM	Household weighted BMI by age/household size (BMI = weight(kg)/square of height (meter))	21.97	3.66	8.53	35.72
Community development	Community development score	1.97	0.72	0.48	3.43
Urban–rural type	1 = urban, 0 = rural	0.41	0.49	0.00	1.00

### 3.3 Model settings

Referring to De Hooge et al. (47), the following model is developed to verify the effect of dietary preference on food waste in residential households:


(1) $$Y_{it}=\beta_{0}+\beta_{1}DP_{it}+\sum k_{it}Z_{it}+\varepsilon_{it}$$

(2) $$Y_{it}=\beta_{0}+\beta_{1}DP_{it}+\beta_{2}RU_{it}+\beta_{3}DP_{it}\times RU_{it}+\sum k_{it}Z_{it}+\varepsilon_{it}$$

where $Y_{it}$ is the explained variable residential household food waste. $DP_{it}$ is the core explanatory variable dietary preference index. $RU_{it}$ is household refrigerator use. $Z_{it}$ is the control variable. $DP_{it}\times RU_{it}$ is the interaction term between dietary preference index and refrigerator use. $\beta_{0},\beta_{1},\beta_{2},\beta_{3},k_{it}$ is the coefficient of the variables, respectively. $\varepsilon_{it}$ is the random error term. i,t is the id of the household head and year, respectively. The impact of dietary preference on food waste is measured using Equation (1), while Equation (2) is employed to test whether refrigerator usage moderates the relationship between dietary preference and food waste.

In terms of specific model selection, since the food waste ratio contains censored data, our study uses a count regression model for the empirical analysis considering the robustness of the estimators.

## 4 Results

### 4.1 Descriptive evidence

**Figure 2:**
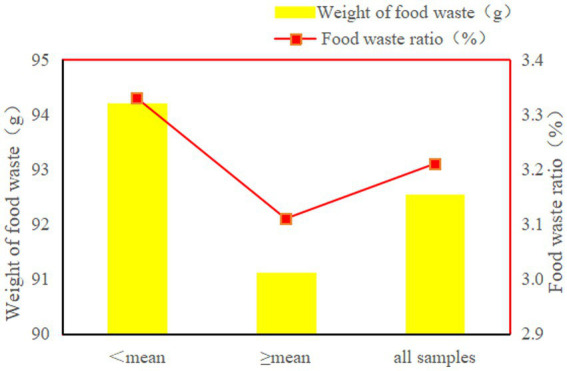
Descriptive analysis.

In order to visualize the link between dietary preference score and residents’ household food waste behavior, a simple comparative analysis of household food waste is first conducted, as shown in Figure 1. It is not difficult to find that the food waste *per capita* for three consecutive days in households (94.21 g) with lower dietary preference score (<3.39) is significantly higher than that in households (91.13 g) with higher dietary preference score (≥3.39). If the food waste ratio is chosen as a comparative indicator, the mean food waste ratio of the sample households is 3.21%. In addition, the food waste ratio of households with lower dietary preference score is 3.33%, which is higher than the sample mean, whereas the food waste ratio of households with higher dietary preference score is the lowest at 3.11%. Thus, Figure 2 provides preliminary evidence that dietary preference influence food waste.

### 4.2 Benchmark regression

According to Equation (1), fitting regression is performed using stepwise regression method, and the result shows (Table 2) that the coefficient of the effect of dietary preference on household food waste is consistently negative and significant at the 5% level after controlling for the possibility of influencing factors at the individual level, the household level, the community level, and the urban–rural level, respectively. Taking column (4) as an example, for every unit increase in dietary preference, food waste of household residents will decrease by three percentage points. This implies that an improvement in dietary preference significantly reduces food waste of household residents, consistent with the findings of Qian et al. (38).

**Table 2:** Dietary preference and food waste behavior.

Variable	Food waste ratio			
	(1)	(2)	(3)	(4)
DP	−0.032^**^ (0.014)	−0.034^**^ (0.014)	−0.032^**^ (0.014)	−0.030^**^ (0.014)
Gender	−0.127^***^ (0.019)	−0.140^***^ (0.020)	−0.146^***^ (0.020)	−0.151^***^ (0.020)
Age	0.001 (0.001)	−0.006^***^ (0.001)	−0.006^***^ (0.001)	−0.006^***^ (0.001)
Education	−0.014 (0.009)	−0.017* (0.009)	−0.006 (0.010)	−0.006 (0.010)
Ethnicity	−0.139^***^ (0.026)	−0.145^***^ (0.026)	−0.129^***^ (0.026)	−0.123^***^ (0.026)
Work status	0.113^***^ (0.019)	0.118^***^ (0.020)	0.108^***^ (0.020)	0.099^***^ (0.020)
Working intensity	0.042^***^ (0.007)	0.050^***^ (0.007)	0.034^***^ (0.008)	0.032^***^ (0.008)
Proportion of families eating out		0.000 (0.000)	0.001 (0.000)	0.001^**^ (0.000)
Family economic condition		0.018^***^ (0.006)	0.021^***^ (0.006)	0.023^***^ (0.006)
Household size		−0.003^*^ (0.006)	−0.005^**^ (0.006)	−0.005^**^ (0.006)
Age 14		−0.003^***^ (0.001)	−0.003^***^ (0.001)	−0.003^***^ (0.001)
Age 60		0.003^***^ (0.000)	0.003^***^ (0.000)	0.003^***^ (0.000)
Family BIM		−0.010^***^ (0.003)	−0.009^***^ (0.003)	−0.010^***^ (0.003)
Community development			−0.067^***^ (0.014)	−0.051^***^ (0.014)
Urban–rural type				−0.066^***^ (0.018)
Region trend	Yes	Yes	Yes	Yes
Time trend	Yes	Yes	Yes	Yes
Constant	1.402^***^ (0.078)	1.816^***^ (0.114)	1.924^***^ (0.116)	1.924^***^ (0.116)
Pseudo *R*^2^	0.043	0.046	0.046	0.047
N	6,253	6,069	6,069	6,069

Standard errors in parentheses; significance level *p < 0.10, **p < 0.05, and ***p < 0.01.

With respect to control variables, significantly more influential on food waste ratio are gender, ethnicity, employment and labor intensity status at the head of household level, income level *per capita* and BMI at the household level, the community development index in which the household is located, and urban–rural type. Specifically, household food waste is more pronounced among female heads of households relative to male, which is consistent with existing results (48). The likely reason for this is that female heads tend to prepare more food based on dietary nutritional balance considerations, with a corresponding higher level of food waste. Ethnicity of the head of household negatively affects the household food waste ratio at a significance level of 1%, with Han households being relatively more frugal with food and having a lower household food waste ratio. It may be related to the more sophisticated cooking styles of the Han. Family with household head in employment and high labor intensity has higher food waste ratio, which is consistent with Wang’s (25) study. A plausible explanation is that the stable source of income allows households to have better expectations of their future lives, and the value that food brings is subjectively weakened. There is a significant positive correlation between logarithmic household income *per capita* and food waste. As household income *per capita* continues to increase, the higher the consumption level and quality of life, the more serious the food waste is. This is consistent with the results of Ding’s (34) study on food waste in Chinese households. Compared to households with lower BMI, the higher the household BMI, the more family members pay attention to figure management. That may increase food waste due to figure control, validating Qian et al. (11). The community development index is calculated based on the scores of the community survey about population density, economic activities, transportation and sanitary conditions, and the entropy method is comprehensively applied. The community development index has a significant negative effect on food waste, verifying Min’s (27) view. As the comprehensive community development index continues to rise, the overall quality of people is also improving. They are more concerned about balanced dietary nutrition, avoiding too much fish and meat, and favoring moderate amounts of staple foods and vegetables, thus reducing food waste. Overall, as both producers and consumers of food, households in rural areas are more prone to food waste than that in urban areas due to their resource endowment advantages and improper storage of crop fruits.

### 4.3 Robust test

#### 4.3.1 Replacement of food waste indicator

In order to verify the robustness of the above regression results, this section selects the logarithm of the weight of food waste as the explained variable drawing on Ding et al. (34) to sequentially examine the effect of dietary preference on the absolute value of food waste. The fitted regression is conducted according to the stepwise regression method. The result shows (Table 3) that dietary preference consistently negatively affects the weight of food waste in residential households, again verifying that the higher the dietary preference score, the lower the food waste.

**Table 3:** Robustness tests I: replacing explained variables.

Variable	Weight of food waste			
	(1)	(2)	(3)	(4)
DP	−0.026^*^ (0.015)	−0.026^*^ (0.015)	−0.027^*^ (0.015)	−0.026^*^ (0.015)
Control variable	Yes	Yes	Yes	Yes
Region trend	Yes	Yes	Yes	Yes
Time trend	Yes	Yes	Yes	Yes
Pseudo *R*^2^	0.023	0.028	0.028	0.028
N	6,169	6,069	6,069	6,069

Standard errors in parentheses; significance level *p < 0.10, **p < 0.05, and ***p < 0.01.

#### 4.3.2 Instrumental variable regression

Households with less food waste are more conscious of saving and healthy eating, resulting in higher dietary preference scores. Conversely, households with more food waste are likely to pay less attention to the concept of healthy eating. That is, there is likely to be a reverse causal link between dietary preference and food waste behavior. To alleviate the possible endogeneity problem, our study tries to introduce an instrumental variable to solve it. Drawing on Wang’s (25) idea of constructing an instrumental variable, our study chooses the provincial dietary preference score as an instrumental variable for the dietary preference of residential households. Logically, this variable is appropriate as an instrumental variable. Firstly, household dietary preference is affected by the environment and culture of the region, and there is an obvious cohort effect. Therefore, the dietary preference at the provincial level is closely related to the dietary preference of household, satisfying the correlation. Secondly, provincial-level dietary preference is not directly related to micro-level residents’ household food waste, thus satisfying exogeneity. The IV-based regression result demonstrates that the *F* value is greater than 10, indicating that the selected instrumental variable is not a weak instrumental variable. Table 4 shows that after effectively controlling for possible endogeneity issue, dietary preference still robustly influences household food waste behavior.

**Table 4:** Robustness tests II: instrumental variable regression.

Variable	Food waste ratio			
	Two-stage regression result			
	(1)	(2)	(3)	(4)
DP	−2.347^***^ (0.858)	−2.837^***^ (1.057)	−2.912^***^ (1.076)	−2.890^***^ (1.089)
Control variable	Yes	Yes	Yes	Yes
	First-stage regression result			
provincial dietary preference	1.067^***^ (0.109)	0.964^***^ (0.116)	1.005^***^ (0.119)	0.993^***^ (0.119)
Control variable	Yes	Yes	Yes	Yes
Weak instrumental variable test	95.52	68.72	71.00	69.41
*N*	6,253	6,069	6,069	6,069

Standard errors in parentheses; significance level *p < 0.10, **p < 0.05, and ***p < 0.01.

### 4.4 Heterogeneity analysis

China is a vast territory with significant geographic and cultural differences. According to the previous analysis, the gender and age characteristics of household decision makers, differences in household size and economic level, urban–rural type, and north–south region also affect household food waste behavior. In order to identify the heterogeneous effects of dietary preference on food waste ratio, our study draws on existing studies (11, 27) to conduct a subgroup comparative analysis from the above perspectives.

#### 4.4.1 Comparative analysis of gender and age of household head

It has been shown that female is significantly different from male in making decision about household food waste due to considerations such as body shape and balanced household dietary. Households headed by females have higher food waste ratio (11, 27). A comparative sub-sample analysis by gender in this sub-section (Table 5) finds that dietary preference significantly reduces female household food waste compared to male-headed household, while other variables remain the same. It is important to pay attention to gender differences and to the ‘Love Food, Hate Waste’ campaign of the female group.

**Table 5:** Heterogeneity analysis: gender.

Variable	Food waste ratio	
	Male	Female
DP	0.019 (0.016)	−0.212^***^ (0.029)
Control variable	Yes	Yes
Region trend	Yes	Yes
Time trend	Yes	Yes
Pseudo *R*^2^	0.045	0.077
*N*	4,978	1,091

Standard errors in parentheses; significance level *p < 0.10, **p < 0.05, and ***p < 0.01.

Famine experience or life experience can affect household members’ food consumption decisions (34). If the difference in food waste ratio between older and younger household heads is measured (Table 6) according to the three criteria of whether it is greater than or equal to the mean, whether it is greater than or equal to 50 years old, and whether it is greater than or equal to 60 years old (25), it is concluded that the food waste ratios of both younger and older household heads decrease brought about by the improvement of their dietary preferences. The higher the dietary preference score, the more emphasis is placed on nutrition and health. As the more reasonable the food consumption structure is, the more choices are made for family meals, and the less food waste is caused. However, the food waste effect of dietary preference for older household heads is not significant. This may be due to their age limitation, resulting in a more concentrated score of dietary preferences with little difference.

**Table 6:** Heterogeneity analysis: age.

Variable	Food waste ratio					
	Whether ≥50		Whether ≥mean		Whether ≥60	
	<50	≥50	<55	≥55	<60	≥60
DP	−0.053^***^ (0.020)	−0.008 (0.019)	−0.034^*^ (0.017)	−0.027 (0.023)	−0.041^**^ (0.016)	−0.026 (0.027)
Control variable	Yes	Yes	Yes	Yes	Yes	Yes
Region trend	Yes	Yes	Yes	Yes	Yes	Yes
Time trend	Yes	Yes	Yes	Yes	Yes	Yes
Pseudo *R*^2^	0.058	0.046	0.050	0.056	0.047	0.068
*N*	2,454	3,615	3,356	2,713	4,178	1891

Standard errors in parentheses; significance level *p < 0.10, **p < 0.05, and ***p < 0.01.

#### 4.4.2 Comparative analysis of household size and household income

Household size can affect collective decision-making rules, which in turn affects the food waste behavior of its members. According to whether the household size is greater than or equal to the mean, the overall samples are divided into small-scale and large-scale households (2). For households of different sizes, the results (Table 7) indicate that an improvement in dietary preference score significantly reduces food waste ratio. In small-scale households, each unit increase in dietary preference will result in a 6.4 percentage point reduction in household food waste ratio. The effect of dietary preference on food waste ratio is not significant for large-scale households. The possible explanation is that large-scale households have more daily meal preparations (11) and the reduction in food waste ratio due to dietary preference is not significant.

**Table 7:** Heterogeneity analysis: household size.

Variable	Food waste ratio	
	Small-scale households	Large-scale households
DP	−0.064^***^ (0.017)	0.019 (0.024)
Control variable	Yes	Yes
Region trend	Yes	Yes
Time trend	Yes	Yes
Pseudo R^2^	0.051	0.063
*N*	3,895	2,174

Standard errors in parentheses; significance level *p < 0.10, **p < 0.05 and ***p < 0.01.

Differences in household economic levels will be reflected in their food purchasing and consumption behaviors (27). It is divided into two sub-samples according to whether or not the economic income *per capita* of the household is greater than or equal to the sample mean. The result of the analysis (Table 8) shows that the dietary preference of high-income households significantly negatively affects food waste ratio, which may be related to the adjustment of dietary structure. When the income level is higher, there is a greater emphasis on healthy eating, a more diverse dietary structure, and reduced food surplus (49). Therefore, the effect of lower food waste ratio brought by higher dietary preference score is more obvious. Comparatively speaking, the decrease in food waste ratio brought about by the increase in dietary preference score of low-income households is not significant.

**Table 8:** Heterogeneity analysis: household income.

Variable	Food waste ratio	
	Low-income households	High-income households
DP	−0.020 (0.027)	−0.038^**^ (0.016)
Control variable	Yes	Yes
Region trend	Yes	Yes
Time trend	Yes	Yes
Pseudo *R*^2^	0.062	0.055
*N*	1,554	4,535

Standard errors in parentheses; significance level *p < 0.10, **p < 0.05, and ***p < 0.01.

#### 4.4.3 Comparative analysis of southerners and northerners

Tracing back to historical origins, there are large differences in the types and structure of cuisine between north and south in China. The group comparison by northern and southern regions (Table 9) reveals increasing the northern families’ dietary preference will significantly reduce food waste at a 1% level compared to southern families. This may be related to the geographical and dietary culture of northern families (11, 49). Compared to the south, the northern region has a stronger emphasis on ‘face’ culture, with larger portion sizes on each plate. If dietary preference can be effectively increased, the effect of food waste reduction will be more obvious in north.

**Table 9:** Heterogeneity analysis: southerners and northerners.

Variable	Food waste ratio	
	South	North
DP	0.017 (0.018)	−0.072^***^ (0.022)
Control variable	Yes	Yes
Region trend	Yes	Yes
Time trend	Yes	Yes
Pseudo *R*^2^	0.073	0.041
*N*	3,327	2,742

Standard errors in parentheses; significance level *p < 0.10, **p < 0.05, and ***p < 0.01.

#### 4.4.4 Comparative analysis of urban–rural type

There are significant differences in the dietary structure of urban and rural residents (50), and food waste behaviors also differ. According to the classification of urban and rural households (Table 10), both can reduce food waste by increasing their dietary preferences. However, the food waste reduction effect of increasing dietary preference among rural households is not significant. A possible explanation for this is that households’ dietary preference scores are more concentrated in rural areas and the effect of higher dietary preference score on food waste is not significant.

**Table 10:** Heterogeneity analysis: urban and rural.

Variable	Food waste ratio	
	Urban	Rural
DP	−0.078^***^ (0.021)	−0.011 (0.018)
Control variable	Yes	Yes
Region trend	Yes	Yes
Time trend	Yes	Yes
Pseudo R^2^	0.065	0.105
N	2,470	3,599

Standard errors in parentheses; significance level *p < 0.10, **p < 0.05, and ***p < 0.01.

## 5 Discussion

### 5.1 Household food waste

In recent years, household food waste has been widely emphasized globally. Scholars at home and abroad have measured the level of household food waste in different countries based on different research methods, and the relevant literature is presented as follows:

As shown in Table 11, food waste in Chinese households is much lower than that in Western households. The difference in the level of food waste may be closely related to the level of economic development (Qian et al., 2021). As a developing country, China’s food losses are mainly due to losses at the production, processing, transportation and storage stages, whereas in most developed countries, where food production and processing technologies are mature, food losses mainly originates from wastage at the retail and consumption stages (56). Another reason for the difference in the level of food waste is the difference in food culture between the East and the West. As a country with a long history, China has a tradition of thrift and frugality since ancient times, advocating for food conservation and opposing food waste.

**Table 11:** Summaries of major studies on food waste in China and abroad.

Author	Country	Main conclusions
Quested et al. (51)	England	Total household waste in the UK is 19% of all food and drink brought home by weight
Silvennoinen et al. (52)	Finland	When comparing the weight of food purchased with the avoidable food waste, the average waste rate is about 4 to 5%
Campoy-Muñoz et al. (53)	Spain	Average household food waste ratio is 42% extrapolated from samples in Spain, Germany, Poland
Yu et al. (54)	America	Households waste an average of 31.9% of their food purchases
Berjan et al. (55)	Serbia	80% of respondents wasted less than 10% of cereals and less than 5% of vegetables and fruits
Author	China	On average, 92.54 g of food is wasted *per capita* in Chinese households on 3 days, with a food waste ratio of 3.21%

### 5.2 The study of moderating effect of refrigerator use

Research has shown that the application of cold storage devices such as refrigerators can help to delay food spoilage relative to ambient conditions, allowing for food preservation periods to be extended (57). Thus, when households are unable to consume leftovers in a timely manner due to preferences or other reasons, households with refrigerators are in a good position to wait for the next consumption by preserving ingredients at low temperatures rather than throwing away the food. Moreover, as refrigerators are upgraded and become increasingly intelligent, the refrigerator system sends food information to the user by checking and tracking the food in real time. This allows households to make informed consumption choices to avoid food being wasted due to spoilage (58).

In order to verify whether refrigerator use has a moderating effect on the effect of dietary preference on food waste, and with reference to the Min et al. (27) study, this section introduces a fitted regression with a interaction term for dietary preference and refrigerator use. Table 12 presents the estimation results of household food waste by step, including variables for dietary preference, refrigerator use, and their cross terms. The estimation of household food waste finds that the interaction term between dietary preference and refrigerator use significantly affects food waste ratio, implying that refrigerator use does have a moderating role in dietary preference influencing food waste behavior.

**Table 12:** Dietary preference, refrigerator use and food waste.

Variable	Food waste ratio			
	(1)	(2)	(3)	(4)
DP	−0.030** (0.014)		−0.024^*^ (0.014)	0.002 (0.021)
RU		−0.153^***^ (0.018)	−0.151^***^ (0.018)	0.002 (0.093)
DP × RU				−0.046^*^ (0.027)
Control variable	Yes	Yes	Yes	Yes
Pseudo *R*^2^	0.047	0.048	0.048	0.049
*N*	6,069	6,067	6,067	6,067

Standard errors in parentheses; significance level *p < 0.10, **p < 0.05, and ***p < 0.01.

Reducing food waste is a complex economic, social and environmental issue (59). Multiple measures should be adhered to reduce food waste, thus achieving the goal of sustainable development. Based on the above studies, it is not difficult to draw the following insights.

Firstly, it is important to publicize knowledge of nutrition and health, raise awareness of food conservation and improve dietary preferences. In order to cultivate healthy dietary habits among urban and rural residents, efforts should be stepped up to publicize nutritional and health information and to popularize the health hazards of poor diets, such as fast food, salty snacks and sugary drinks. At the same time, easy-to-understand posters and pamphlets should be designed to continue the ‘Clean Your Plate Campaign’ for raising awareness of food conservation among urban and rural residents.

Secondly, it is important for developing countries in general to accelerate the promotion of refrigerators to reduce food waste. Households without refrigerators can be subsidized for home appliances to encourage the purchase of refrigerators, which together will help to save food and reduce waste. Given the varied financial situations of different demographics regarding the purchase of refrigerators, appliance subsidy policies for refrigerators should be tilted toward low-income families and rural groups.

Finally, it is crucial to focus on improving the dietary preference of small-scale households. In view of the fact that an increase in the dietary preference score of small-sized families will have a greater effect on reducing food waste and the reality that China’s current family structure is gradually shifting toward small-sized families, attention should be focused on the dietary preference of small-sized family members. Regular family communication and exchange can be organized to understand their dietary situations and confusions, and corresponding support and suggestions can be provided to improve the overall dietary preference score of families.

It should be noted that our study has certain shortcomings. Firstly, the research data on household food waste among Chinese residents is not novel enough. The investigation on household food waste is time-consuming and laborious, thus data collection is difficult. The existing databases on food waste at the household level are very limited. The accessible CHNS database collects data on food waste at the household level, but the data for our study is outdated due to the discontinuation of food waste data in 2015 and beyond. However, the perspective of our study is the impact of dietary preferences on food waste. Considering the millennia-old tradition of food consumption and the current state of household dietary preference, it is expected that our study can still provide credible and contemporary findings. Secondly, although higher dietary preference score has a certain effect on food waste, dietary preference is strongly influenced by geography and socio-culture. How to improve the residents’ dietary preference is an urgent problem that needs to be solved. Further research on the influencing factors of the residents’ dietary preference needs to be followed up.

## 6 Conclusion

Based on the CHNS database, our study examines the association between dietary preference and food waste in Chinese urban and rural households. Statistical descriptions reveal that 63% of the households exhibit varying degrees of food waste behavior, indicating that food waste is relatively common. The empirical results show that:

- There is a significant correlation between dietary preference and food waste. Overall, increasing dietary preference score will significantly reduce household food waste among residents.
- Heterogeneity analysis reveals that dietary preference has a more pronounced effect on reducing household food waste in female-headed households relative to male-headed households. The food waste reduction effect of dietary preference is more significant for households with younger household heads compared to households with older household heads. Given the amount of food preparation for different household sizes, the food waste reduction effect of dietary preference is more pronounced for smaller households. In addition, dietary preference has a significant negative impact on food waste in urban households.
- Further analysis reveals that the moderating effect of refrigerator use on the effect of dietary preference on food waste is further enhanced by household owning a refrigerator, grouped according to whether or not the household owns a refrigerator.

## Data availability statement

The raw data supporting the conclusions of this article will be made available by the authors, without undue reservation.

## Ethics statement

Written informed consent was obtained from the individual(s), and minor(s)’ legal guardian/next of kin, for the publication of any potentially identifiable images or data included in this article.

## Author contributions

LZ: Writing – review & editing, Writing – original draft, Methodology, Funding acquisition, Data curation. LY: Writing – review & editing, Supervision, Funding acquisition, Data curation, Conceptualization. LQ: Writing – review & editing, Visualization, Validation, Supervision, Data curation, Conceptualization. XZ: Writing – review & editing, Validation, Supervision, Formal analysis, Data curation.

## Appendix

**Table A1:** Food preferences.

Food item How much do you like this food: Like very much, like somewhat, dislike somewhat, or dislike very much?	1 dislike very much2 dislike 3 neutral4 like 5 like very much9 does not eat this food
Fast food (KFC, pizza, hamburgers, etc.)	
Salty snack foods (potato chips, pretzels, French fries, etc.)	
Fruit	
Vegetables	
Soft drinks and sugared fruit drinks	

## References

[1] Khalid S Malik AU Ullah MI Khalid MS Javeed HMR Naeem MA. Food waste: causes and economic losses estimation at household level in Pakistan. Environ Sci Pollut Res. (2023) 30:99284–97. doi: 10.1007/s11356-023-28775-4, PMID: 37632618

[2] Li YY Wang LE Liu G Cheng S. Rural household food waste characteristics and driving factors in China. Resour Conserv Recycl. (2021) 164:105209. doi: 10.1016/j.resconrec.2020.105209

[3] Tonini D Albizzati PF Astrup TF. Environmental impacts of food waste: learnings and challenges from a case study on UK. Waste Manag. (2018) 76:744–66. doi: 10.1016/j.wasman.2018.03.032, PMID: 29606533

[4] United Nations Environment Programme (2024). Food waste index report 2024. Think Eat Save: Tracking Progress to Halve Global Food Waste. Available at: https://wedocs.unep.org/20.500.11822/45230

[5] Boliko MC. FAO and the situation of food security and nutrition in the world. J Nutr Sci Vitaminol. (2019) 65:S4–8. doi: 10.3177/jnsv.65.S4, PMID: 31619643

[6] Principato L Mattia G di Leo A Pratesi CA. The household wasteful behaviour framework: a systematic review of consumer food waste. Ind Mark Manag. (2021) 93:641–9. doi: 10.1016/j.indmarman.2020.07.010

[7] Song G Semakula HM Fullana PP. Chinese household food waste and its’climatic burden driven by urbanization: a Bayesian belief network modelling for reduction possibilities in the context of global efforts. J Clean Prod. (2018) 202:916–24. doi: 10.1016/j.jclepro.2018.08.233

[8] Thyberg KL Tonjes DJ. Drivers of food waste and their implications for sustainable policy development. Resources Conserv Recyc. (2016) 106:110–23. doi: 10.1016/j.resconrec.2015.11.016

[9] Kummu M De Moel H Porkka M. Lost food, wasted resources: global food supply chain losses and their impacts on freshwater, cropland, and fertiliser use. Sci Total Environ. (2012) 438:477–89. doi: 10.1016/j.scitotenv.2012.08.092, PMID: 23032564

[10] FAO. The state of food and agriculture, moving forward on food loss and waste reduction. Rome: Food and Agriculture Organization of the United Nations (2019) http://www.fao.org/publications/sofa/en/.

[11] Qian L Li F Liu H Wang L. Are the slimmer more wasteful? The correlation between body mass index and food wastage among Chinese youth. Sustain For. (2022) 14:1411. doi: 10.3390/su14031411

[12] Qian L Rao Q Liu H McCarthy B Liu LX Wang L. Food waste and associated carbon footprint: evidence from Chinese universities. Ecosyst Health Sustain. (2022) 8:2130094. doi: 10.1080/20964129.2022.2130094

[13] Oehman JM Babbitt CW Flynn C. What predicts and prevents source separation of household food waste? An application of the theory of planned behavior. Resour Conserv Recycl. (2022) 186:106492. doi: 10.1016/j.resconrec.2022.106492

[14] Conrad Z. Daily cost of consumer food wasted, inedible, and consumed in the United States, 2001–2016. Nutr J. (2020) 19:35. doi: 10.1186/s12937-020-00552-w, PMID: 32306976 PMC7168972

[15] Caldeira C de Laurentiis V Corrado S van Holsteijn F Sala S. Quantification of food waste per product group along the food supply chain in the European Union: a mass flow analysis. Resour Conserv Recycl. (2019) 149:479–88. doi: 10.1016/j.resconrec.2019.06.011, PMID: 31582876 PMC6703187

[16] Ammann J Osterwalder O Siegrist M Hartmann C Egolf A. Comparison of two measures for assessing the volume of food waste in Swiss households. Resour Conserv Recycl. (2021) 166:105295. doi: 10.1016/j.resconrec.2020.105295

[17] Ananda J Gayana Karunasena G Pearson D. Identifying interventions to reduce household food waste based on food categories. Food Policy. (2022) 111:102324. doi: 10.1016/j.foodpol.2022.102324

[18] Li X Jiang Y Qing P. Estimates of household food waste by categories and their determinants: evidence from China. Food Secur. (2023) 12:776. doi: 10.3390/foods12040776, PMID: 36832853 PMC9956864

[19] Ananda J Karunasena GG Mitsis A Kansal M Pearson D. Analysing behavioural and socio-demographic factors and practices influencing Australian household food waste. J Clean Prod. (2021) 306:127280. doi: 10.1016/j.jclepro.2021.127280

[20] Van Bemmel A Parizeau K. Is it food or is it waste? The materiality and relational agency of food waste across the value chain. J Cult Econ. (2020) 13:207–20. doi: 10.1080/17530350.2019.1684339

[21] Hermanussen H Loy J-P. Household food waste: a meta-analysis. Environmental Challenges. (2024) 14:100809. doi: 10.1016/j.envc.2023.100809

[22] Nahman A de Lange W Oelofse S Godfrey L. The costs of household food waste in South Africa. Waste Manag. (2012) 32:2147–53. doi: 10.1016/j.wasman.2012.04.012, PMID: 22608682

[23] Kansal M Ananda J Mitsis A Karunasena GG Pearson D. Food waste in households: children as quiet powerhouses. Food Qual Prefer. (2022) 98:104524. doi: 10.1016/j.foodqual.2021.104524

[24] Hu Y Zhou YH Han YJ. Resources and economic effects analysis of reducing food waste. China Popul Resour Environ. (2013) 23:150–5.

[25] Wang Z Jiang J Zeng Q. The effect of dietary awareness on household food waste. Waste Manag Res. (2023) 41:164–72. doi: 10.1177/0734242X221105435, PMID: 35723618

[26] Wang R Lu S Zhou L Yang Z Tang Z Zhao M. Assessing nutritional and economic aspects of food loss and waste in China. Sustain Product Consum. (2023) 42:95–105. doi: 10.1016/j.spc.2023.09.010

[27] Min S Wang XB Yu XH. Does dietary knowledge affect household food waste in the developing economy of China? Food Policy. (2021) 98:101896. doi: 10.1016/j.foodpol.2020.101896

[28] Cheng XY. Research on the influence of information intervention on household food waste behavior. Tianjin: Tianjin University of Science and Technology (2021).

[29] Secondi L Principato L Laureti T. Household food waste behaviour in EU-27 countries: a multilevel analysis. Food Policy. (2015) 56:25–40. doi: 10.1016/j.foodpol.2015.07.007

[30] Wu Y Tian X Li X Yuan H Liu G. Characteristics, influencing factors, and environmental effects of plate waste at university canteens in Beijing. China Resour Conserv Recycl. (2019) 149:151–9. doi: 10.1016/j.resconrec.2019.05.022

[31] Elhoushy S Jang SC. Religiosity and food waste reduction intentions: a conceptual model. Int J Consum Stud. (2021) 45:287–302. doi: 10.1111/ijcs.12624

[32] Szabó-Bódi B Kasza G Szakos D. Assessment of household food waste in Hungary. Br Food J. (2018) 120:625–38. doi: 10.1108/BFJ-04-2017-0255

[33] Piras S. Community social capital and status: the social dilemma of food waste. Ecol Econ. (2021) 183:106954. doi: 10.1016/j.ecolecon.2021.106954

[34] Ding Y Min S Wang XB Yu X. Memory of famine: the persistent impact of famine experience on food waste behavior. China Econ Rev. (2022) 73:101795. doi: 10.1016/j.chieco.2022.101795

[35] Graham-Rowe E Jessop DC Sparks P. Predicting household food waste reduction using an extended theory of planned behaviour. Resour Conserv Recycl. (2015) 101:194–202. doi: 10.1016/j.resconrec.2015.05.020

[36] Quested T Ingle R Parry A (2013) Household food and drink waste in the United Kingdom 2012. London: WRAP 3–134.

[37] Holsteijn FV Kemna R. Minimizing food waste by improving storage conditions in household refrigeration. Resour Conserv Recycl. (2018) 128:25–31. doi: 10.1016/j.resconrec.2017.09.012

[38] Qian L Li F Qian Z. Factors affecting food waste in college canteens. Resources Sci. (2019) 41:1859–70. doi: 10.18402/resci.2019.10.09

[39] Mattar L Abiad MG Chalak A Diab M Hassan H. Attitudes and behaviors shaping household food waste generation: lessons from Lebanon. J Clean Prod. (2018) 198:1219–23. doi: 10.1016/j.jclepro.2018.07.085

[40] Tang DX Wang Q. Diet antropological study of food waste problem in Chinese dining Table. J Qinghai Minzu Univ. (2021) 47:1–10.

[41] Porpino G Wansink B Parente J. Wasted Positive Intentions: The Role of Affection and Abundance on Household Food Waste. Journal of Food Products Marketing. (2016) 22:733–751. doi: 10.1080/10454446.2015.1121433

[42] Wharton FG Foth M Hee J. Identifying factors that promote consumer behaviours causing expired domestic food waste. J Consum Behav. (2014) 13:393–402. doi: 10.1002/cb.1488

[43] Yildirim H Roberto C. Food wastage in Turkey:an exploratory survey on household food waste. J Food Nutr Res. (2016) 4:229–41.

[44] FAO. Food and agriculture Organization of the United Nations. Guidelines for measuring household and individual dietary diversity. Rome: Food and Agriculture Organization (2011).

[45] Boiteau JM Pingali P. Can we agree on a food loss and waste definition? An assessment of definitional elements for a globally applicable framework. Glob Food Sec. (2023) 37:100677. doi: 10.1016/j.gfs.2023.100677

[46] Fami HS Aramyan LH Sijtsema SJ Alambaigi A. Determinants of household food waste behavior in Tehran city: a structural model. Resour Conserv Recycl. (2019) 143:154–66. doi: 10.1016/j.resconrec.2018.12.033

[47] de Hooge IE Oostindjer M Aschemann-Witzel J Normann A Loose SM Almli VL. This apple is too ugly for me! Food Qual Prefer. (2017) 56:80–92. doi: 10.1016/j.foodqual.2016.09.012

[48] Buzby JC Guthrie JF. Plate waste in school nutrition programs. J Consum Aff. (2002) 36:220–38. doi: 10.1111/j.1745-6606.2002.tb00431.x

[49] Xu Z Zhang Z Liu H Zhong F Bai J Cheng S. Food-away-from-home plate waste in China: preference for variety and quantity. Food Policy. (2020) 97:101918. doi: 10.1016/j.foodpol.2020.101918

[50] Liu C Shang J Liu C Wang H Wang S. Policy recommendations for reducing food waste: an analysis based on a survey of urban and rural household food waste in Harbin. China Sustainability. (2023) 15:11122. doi: 10.3390/su151411122

[51] Quested T Parry A. New estimates for household food and drink waste in the UK. England: Waste & Reources Action Programme (WRAP) (2011).

[52] Silvennoinen K Katajajuuri J-M Hartikainen H Heikkilä L Reinikainen A. Food waste volume and composition in Finnish households. Br Food J. (2014) 116:1058–68. doi: 10.1108/BFJ-12-2012-0311

[53] Campoy-Muñoz P Cardenete M Delgado M. Economic impact assessment of food waste reduction on European countries through social accounting matrices. Resour Conserv Recycl. (2017) 122:202–9. doi: 10.1016/j.resconrec.2017.02.010

[54] Yu Y Jaenicke EC. Estimating food waste as household production inefficiency. Am J Agric Econ. (2020) 102:525–47. doi: 10.1002/ajae.12036

[55] Berjan S Vaško Ž Ben Hassen T el Bilali H Allahyari MS Tomić V. Assessment of household food waste management during the COVID-19 pandemic in Serbia: a cross-sectional online survey. Environ Sci Pollut Res. (2022) 29:11130–41. doi: 10.1007/s11356-021-16485-8, PMID: 34532805 PMC8445639

[56] Parfitt J Barthel M Macnaughton S. Food waste within food supply chains: quantification and potential for change to 2050. Philos Trans Roy Soc B: Biol Sci. (2010) 365:3065–81. doi: 10.1098/rstb.2010.0126, PMID: 20713403 PMC2935112

[57] Davenport ML Qi D Roe BE. Food-related routines, product characteristics, and household food waste in the United States: a refrigerator-based pilot study. Resources Conserv Recycl. (2019) 150:104440. doi: 10.1016/j.resconrec.2019.104440

[58] Nasir H Aziz W B W Ali F (2018) The implementation of IoT based smart refrigerator system. In 2nd international conference on smart sensors and application (ICSSA). Kuching: IEEE 48–52.

[59] Ponis ST Papanikolaou P-A Katimertzoglou P Ntalla AC Xenos KI. Household food waste in Greece: a questionnaire survey. J Clean Prod. (2017) 149:1268–77. doi: 10.1016/j.jclepro.2017.02.165

